# Improving representation of ethnic minorities in psychiatric research

**DOI:** 10.1192/bjb.2025.10118

**Published:** 2025-08

**Authors:** Rithik Gaikwad

**Affiliations:** University of Manchester, Manchester, UK

As a medical student with a growing interest in psychiatry, I’ve become aware of the limited representation of Black, Asian and minority ethnic individuals in psychiatric research. This gap doesn’t just affect data quality, it also has a direct impact on how fairly and effectively care is delivered across diverse populations.

A recent article by Onozawa et al explored the challenges faced by Black and South Asian women when accessing perinatal mental health services, highlighting issues including cultural stigma, language barriers and the lack of culturally appropriate support.^1^ Although this work is encouraging, it also sheds light on a wider issue: many studies in psychiatry either include very few participants from ethnic minority groups or neglect to report ethnicity altogether.

This is a real concern. As students, we are taught to rely on research to guide our practice. But if the research doesn’t reflect the diversity of the population, how can we be confident we’re delivering evidence-based care to everyone? This is particularly important in psychiatry, in which an individual’s cultural background can significantly influence symptom presentation, service engagement and treatment responses, emphasising the need for data that accurately reflect the diversity of real-world populations.

I’ve also observed that ethnicity in research is sometimes recorded in vague and overly broad terms such as ‘non-White’ or ‘Other’.^2^ These labels offer little insight and risk overlooking meaningful differences between groups. Without more precise and consistent reporting, we lose valuable opportunities to understand real inequalities – and to appreciate the cultural nuances that shape mental health experiences.

Strengthening the inclusivity of our evidence base requires more than improved recruitment alone, it calls for deeper community engagement and clear, consistent standards for how ethnicity is defined and reported. This responsibility does not rest solely with researchers; journals, funding bodies, academic institutions and even medical students all have important parts to play.

For psychiatry to deliver fair and effective care to all, it must better reflect the full diversity of the populations it represents. This begins with ensuring that research includes participants from all backgrounds.
